# Probabilistic linear widths of Sobolev space with Jacobi weights on $[-1,1]$

**DOI:** 10.1186/s13660-017-1540-7

**Published:** 2017-10-23

**Authors:** Xuebo Zhai, Xiuyan Hu

**Affiliations:** School of Mathematical and Statistics, Zaozhuang University, Zaozhuang, 277160 China

**Keywords:** 41A46, 41A25, 28C20, 42C15, probabilistic linear widths, Jacobi weights, weighted Sobolev classes, Gaussian measure

## Abstract

Optimal asymptotic orders of the probabilistic linear $(n,\delta)$-widths of $\lambda_{n,\delta }(W^{r}_{2,\alpha,\beta }, \nu,L_{q,\alpha,\beta })$ of the weighted Sobolev space $W_{2,{\alpha, \beta }}^{r}$ equipped with a Gaussian measure *ν* are established, where $L_{q,\alpha,\beta }$, $1\leq q\leq \infty $, denotes the $L_{q}$ space on $[-1,1]$ with respect to the measure $(1-x)^{\alpha }(1+x)^{\beta }$, $\alpha,\beta > -1/2$.

## Introduction

This paper mainly focuses on the study of probabilistic linear $(n,\delta)$-widths of a Sobolev space with Jacobi weights on the interval $[-1,1]$. This problem has been investigated only recently. For calculation of probabilistic linear $(n,\delta)$-widths of the Sobolev spaces equipped with Gaussian measure, we refer to [1–5]. Let us recall some definitions.

Let *K* be a bounded subset of a normed linear space *X* with the norm $\Vert \cdot \Vert _{X}$. The linear *n*-width of the set *K* in *X* is defined by

$$\lambda_{n}(K,X)= \inf_{L_{n}}\sup _{x\in K} \Vert x-L_{n}x\Vert _{X}, $$

where $L_{n}$ runs over all linear operators from *X* to *X* with rank at most *n*.

Let *W* be equipped with a Borel field $\mathcal{B}$ which is the smallest *σ*-algebra containing all open subsets. Assume that *ν* is a probability measure defined on $\mathcal{B}$. Let $\delta \in [0,1)$. The probabilistic linear $(n,\delta)$-width is defined by

$$\lambda_{n,\delta }(W,\nu, X)=\inf_{G_{\delta }} \lambda_{n}(W\backslash G_{\delta },X), $$

where $G_{\delta }$ runs through all possible *ν*-measurable subsets of *W* with measure $\nu (G_{\delta })\leq \delta $. Compared with the classical case analysis (see [2] or [6]), the probabilistic case analysis, which reflects the intrinsic structure of the class, can be understood as the *ν*-distribution of the approximation on all subsets of *W* by *n*-dimensional subspaces and linear operators with rank *n*.

In his recent paper [7], Wang has obtained the asymptotic orders of probabilistic linear $(n,\delta)$-widths of the weighted Sobolev space on the ball with a Gaussian measure in a weighted $L_{q}$ space. Motivated by Wang’s work, this paper considers the probabilistic linear $(n,\delta)$-widths on the interval $[-1,1]$ with Jacobi weights and determines the asymptotic orders of the probabilistic linear $(n,\delta)$-widths. The difference between the work of Wang and ours lies in the different choices of the weighted points for the proofs of discretization theorems.

## Main results

Consider the Jacobi weights

$$w_{\alpha,\beta }(x):=(1-x)^{\alpha }(1+x)^{\beta }, \quad \alpha, \beta > -1/2. $$

Denote by $L_{p,\alpha,\beta }\equiv L_{p}(w_{\alpha,\beta })$, $1 \le p<\infty $, the space of measurable functions defined on $[-1,1]$ with the finite norm

$$\Vert f\Vert _{p,\alpha,\beta }:= \biggl(\int_{-1}^{1}\bigl\vert f(x)\bigr\vert ^{p} w_{\alpha, \beta }(x)\,dx \biggr)^{1/p}, \quad 1\le p< \infty, $$

and for $p=\infty $ we assume that $L_{\infty,\alpha,\beta }$ is replaced by the space $C[-1,1]$ of continuous functions on $[-1,1]$ with the uniform norm. Let $\Pi_{n}$ be the space of all polynomials of degree at most *n*. Denote by $\mathbb{P}_{n}$ the space of all polynomials of degree *n* which are orthogonal to polynomials of low degree in $L_{2}(w_{\alpha,\beta })$. It is well known that the classical Jacobi polynomials $\{P_{n}^{(\alpha,\beta)}\}_{n=0}^{\infty }$ form an orthogonal basis for $L_{2,\alpha,\beta }:=L_{2}([-1,1],w_{\alpha, \beta })$ and are normalized by $P_{n}^{(\alpha,\beta)}(1)= \bigl({\scriptsize\begin{matrix}{}n+\alpha \cr n\end{matrix}}\bigr)$ (see [8]). In particular,

$$\int_{-1}^{1} P_{n}^{(\alpha,\beta)}(x)P_{n}^{(\alpha,\beta)}(y)w _{\alpha,\beta }(x)\,dx=\delta_{n,m}h_{n}{(\alpha,\beta)}, $$

where

$$h_{n}{(\alpha,\beta)}=\frac{\Gamma (\alpha +\beta +2)}{\Gamma (\alpha +1)\Gamma (\beta +1)} \frac{\Gamma (n+\alpha +1)\Gamma (n+ \beta +1)}{(2n+\alpha +\beta +1)\Gamma (n+1)\Gamma (n+\alpha +\beta +1)} \sim n^{-1} $$

with constants of equivalence depending only on *α* and *β*. Then the normalized Jacobi polynomials $P_{n}(x)$, defined by

$$P_{n}(x)=\bigl(h_{n}^{(\alpha,\beta)}\bigr)^{-1/2}P_{n}^{(\alpha,\beta)}(x),\quad n=0,1,\ldots, $$

form an orthonormal basis for $L_{2,\alpha,\beta }$, where the inner product is defined by

$$\langle f,g\rangle:= \int_{-1}^{1} f(x) \overline{g(x)}w_{\alpha, \beta }(x)\,dx. $$

Denote by $S_{n}$ the orthogonal projector of $L_{2}(w_{\alpha, \beta })$ onto $\Pi_{n}$ in $L_{2}(w_{\alpha,\beta })$, which is called the Fourier partial summation operator. Consequently, for any $f\in L_{2}(W_{\alpha,\beta })$,

2.1 $$ f=\sum_{l=0}^{\infty }\langle f,P_{l}\rangle P_{l} ,\qquad S_{n}f:=\sum _{l=0}^{n} \langle f,P_{l}\rangle P_{l} . $$

It is well known that (see Proposition 1.4.15 in [9]) $P_{n}^{(\alpha,\beta)}$ is just the eigenfunction corresponding to the eigenvalues $-n(n+\alpha +\beta +1)$ of the second-order differential operator

$$D_{\alpha,\beta }:= \bigl(1-x^{2}\bigr)D^{2}-\bigl(\alpha -\beta +(\alpha +\beta +2)x\bigr)D, $$

which means that

$$D_{\alpha,\beta }P_{n}^{(\alpha,\beta)}(x)=-n(n+\alpha +\beta +1)P _{n}^{(\alpha,\beta)}(x). $$

Given $r>0$, we define the fractional power $(-D_{\alpha,\beta })^{r/2}$ of the operator $-D_{\alpha,\beta }$ on *f* by

$$(-D_{\alpha,\beta })^{r/2} (f)= \sum_{k=0}^{\infty } \bigl(k(k+\alpha + \beta +1)\bigr)^{r/2} \langle f,P_{k} \rangle P_{k}, $$

in the sense of distribution. We call $f^{(r)}:=(-D_{\alpha,\beta })^{r/2}$ the *r*th order derivative of the distribution *f*. It then follows that for $f\in L_{2,\alpha,\beta } $, $r\in R$, the Fourier series of the distribution $f^{(r)}$ is

$$f^{(r)}=\sum_{k=1}^{\infty } \bigl(k(k+\alpha +\beta +1)\bigr)^{r/2}\langle f,P _{k}\rangle P_{k}. $$

Using this operator, we define the weighted Sobolev class as follows: For $r>0$ and $1\le p\le \infty $,

$$W_{p,\alpha,\beta }^{r}\bigl([-1,1]\bigr)\equiv W_{p,{\alpha,\beta }}^{r}:= \bigl\{ f\in L_{p,\alpha,\beta } : \Vert f\Vert _{W_{p,\alpha,\beta }^{r}}:= \Vert f \Vert _{p,\alpha,\beta }+\bigl\Vert (-D_{\alpha,\beta })^{\frac{r}{2}}(f)\bigr\Vert _{p,\alpha,\beta }< \infty \bigr\} , $$

while the weighted Sobolev class $BW_{p,{\alpha,\beta }}^{r}$ is defined to be the unit ball of $W_{p,{\alpha,\beta }}^{r}$. When $p=2$, the norm $\Vert \cdot \Vert _{ W_{2,\alpha,\beta }^{r}}$ is equivalent to the norm $\Vert \cdot \Vert _{\overline{W}_{2,\alpha,\beta }^{r}}$, and we can rewrite $W_{2,\alpha,\beta }^{r}$ as

$$\begin{aligned} W_{2,\alpha,\beta }^{r} =&\overline{W}_{2,\alpha,\beta }^{r} \\ :=& \Biggl\{ f(x) =\sum_{l=0}^{\infty }\langle f,P_{n}\rangle P_{n}(x): \Vert f\Vert _{\overline{W}_{2,\alpha,\beta }^{r}}^{2}:=\langle f,P_{0}\rangle ^{2}+\bigl\langle f^{(r)},f^{(r)}\bigr\rangle \\ &= \langle f,P_{0}\rangle^{2}+\sum _{k=1}^{\infty } \bigl(k(k+\alpha + \beta +1) \bigr)^{r}\langle f,P_{k}\rangle^{2}< \infty \Biggr\} \end{aligned}$$

with the inner product

$$\langle f,g\rangle_{r}:=\langle f,P_{0}\rangle \langle g,P_{0}\rangle +\bigl\langle f^{(r)},g^{(r)}\bigr\rangle . $$

Obviously, $\overline{W}_{2,\alpha,\beta }^{r}$ is a Hilbert space. We equip $\overline{W}_{2,\alpha,\beta }^{r}=W_{2,\alpha,\beta }^{r} $ with a Gaussian measure *ν* whose mean is zero and whose correlation operator $C_{\nu }$ has eigenfunctions $P_{l}(x)$, $l=0,1,2,\dots $, and eigenvalues

$$\lambda_{0}=1,\qquad \lambda_{l}=\bigl(l(l+\alpha +\beta +1) \bigr)^{-s/2} ,\quad l=1,2, \dots, s>1, $$

that is,

$$C_{\nu }P_{0}=P_{0},\qquad C_{\nu }P_{l}= \lambda_{l} P_{l}, \quad l=1,2, \dots. $$

Then (see [10], pp.48-49),

$$\langle C_{\nu }f,g\rangle_{r}= \int_{\overline{W}_{2,{\alpha,\beta }}^{r}} \langle f,h\rangle_{r} \langle g,h \rangle_{r} \nu (dh). $$

By Theorem 2.3.1 of [10] the Cameron-Martin space $H(\nu)$ of the Gaussian measure *ν* is $\overline{W}_{2,{\alpha,\beta }}^{r+s/2}$, i.e.,

$$H(\nu)= \overline{W}_{2,{\alpha,\beta }}^{r+s/2}. $$

See [10] and [11] for more information about the Gaussian measure on Banach spaces.

Throughout the paper, $A(n,\delta)\asymp B(n,\delta)$ means $A(n,\delta)\ll B(n,\delta)$ and $A(n,\delta)\gg B(n,\delta)$, $A(n,\delta)\ll B(n,\delta)$ means that there exists a positive constant *c* independent of *n* and *δ* such that $A(n,\delta) \le cB(n,\delta)$. If $1\le q\le \infty$, $r>(2+2\min \{0,\max \{ \alpha,\beta \}\})(1/p-1/q)_{+}$, the space $W_{p,\alpha,\beta } ^{r}$ can be continuously embedded into the space $L_{q,{\alpha, \beta }}$ (see Lemma 2.3 in [12]).

Set $\rho =r+\frac{s}{2}$. The main result of this paper can be formulated as follows.

### Theorem 2.1

*Let* $1\le q \le \infty$, $\delta \in (0,1/2] $, *and let* $\rho >1/2+(2\max \{\alpha,\beta \}+1)(1/2+1/q)_{+}$. *Then*

2.2 $$ \lambda_{n,\delta }\bigl(W_{2,\alpha,\beta }^{r},\nu,L_{q,\alpha,\beta }\bigr)\asymp \textstyle\begin{cases} n^{1/2-\rho }(1+n^{-\min \{1/2,1/q\}})(\ln (\frac{1}{\delta }))^{ \frac{1}{2}}, & 1\leq q< \infty, \\ n^{1/2-\rho }(\ln (\frac{n}{\delta }))^{\frac{1}{2}}, & q=\infty. \end{cases} $$

For the proof of Theorem 2.1, the discretization technique is used (see [1, 4, 13, 14]). Since the known results of the probabilistic linear widths of the identity matrix on $\mathbb{R}^{m}$ are inappropriate here, the probabilistic linear widths of diagonal matrixes on $\mathbb{R}^{m}$ are adopted for the proof of the upper estimates.

## Main lemmas

Let $\ell_{q}^{m}$ ($1\le q\le \infty$) denote the space $\mathbb{R}^{m}$ equipped with the $\ell_{q}^{m}$-norm defined by

$$\Vert x\Vert _{\ell_{q}^{m}}:= \textstyle\begin{cases} (\sum_{i=1}^{m} \vert x_{i}\vert ^{q} )^{\frac{1}{q}}, &1\le q< \infty, \\ \max_{1\le i\le m} \vert x_{i}\vert , &q=\infty. \end{cases} $$

We identify $\mathbb{R}^{m}$ with the space $\ell_{2}^{m}$, denote by $\langle x,y\rangle $ the Euclidean inner product of $x,y\in \mathbb{R} ^{m}$, and write $\Vert \cdot \Vert _{2}$ instead of $\Vert \cdot \Vert _{\ell_{2} ^{m}}$.

Consider in $\mathbb{R}^{m}$ the standard Gaussian measure $\gamma_{m}$, which is given by

$$\gamma_{m}(G)=(2\pi)^{-m/2} \int_{G} \exp^{\frac{-\Vert x\Vert ^{2}}{2}}\,dx, $$

where *G* is any Borel subset in $\mathbb{R}^{m}$. Let $1\le q\le \infty $, $1\le n< m$, and $\delta \in [0,1)$. The probabilistic linear $(n,\delta)$-width of a linear mapping $T:\mathbb{R}^{m}\rightarrow l ^{m}_{q}$ is defined by

$$\lambda_{n,\delta }\bigl(T:\mathbb{R}^{m}\rightarrow l^{m}_{q},\gamma_{m}\bigr)= \inf _{G_{\delta }}\inf_{T_{n}}\sup_{\mathbb{R}^{m}\setminus G_{\delta }} \Vert Tx-T_{n}x\Vert _{l_{q}^{m}}, $$

where $G_{\delta }$ runs over all possible Borel subsets of $\mathbb{R}^{m}$ with measure $\gamma_{m}(G_{\delta })\leq \delta $, and $T_{n}$ runs over all linear operators from $\mathbb{R}^{m}$ to $l_{q}^{m}$ with rank at most *n*.

Throughout the paper, *D* denotes the $m\times m$ real diagonal matrix $\operatorname{diag}(d_{1},\ldots,d_{m})$ with $d_{1}\geq d_{2}\ge \cdots \ge d_{m}>0$, $D_{n}$ denotes the $m\times m$ real diagonal matrix $\operatorname{diag}(d_{1},\ldots,d_{n},0,\ldots,0)$ with $1\le n\le m$, and $I_{m}$ denotes the $m\times m$ identity matrix. Moreover, $\{e_{1},\ldots,e_{m}\}$ denotes the standard orthonormal basis in $\mathbb{R}^{m}$:

$$e_{1}=(1,0,\ldots,0),\qquad \ldots,\qquad e_{m}=(0,\ldots,0,1). $$

Now, we introduce several lemmas which will be used in the proof of Theorem 2.1.

### Lemma 3.1

1. (*See* [1]) *If* $1\le q\le 2$, $m\ge 2n$, $\delta \in (0,1/2]$, *then*
  3.1 $$ \lambda_{n,\delta }\bigl(I_{m}:\mathbb{R}^{m} \rightarrow l^{m}_{q},\gamma_{m}\bigr) \asymp m^{1/q}+m^{1/q-1/2}\sqrt{\ln (1/\delta)}. $$
2. (*See* [4]) *If* $2\le q<\infty $, $m\ge 2n$, $\delta \in (0,1/2]$, *then*
  3.2 $$ \lambda_{n,\delta }\bigl(I_{m}:\mathbb{R}^{m} \rightarrow l^{m}_{q},\gamma_{m}\bigr) \asymp m^{1/q}+\sqrt{\ln (1/\delta)}. $$
3. (*See* [5]) *If* $q=\infty $, $m\ge 2n$, $\delta \in (0,1/2]$, *then*
  3.3 $$ \lambda_{n,\delta }\bigl(I_{m}:\mathbb{R}^{m} \rightarrow l^{m}_{q},\gamma_{m}\bigr) \asymp \sqrt{\ln \bigl((m-n)/\delta }\bigr)\asymp \sqrt{\ln m+\ln (1/\delta)}. $$

### Lemma 3.2

(See [7])

*Assume that*

$$\sum_{i=1}^{m}d_{i}^{\beta } \le C(m,\beta)\quad \textit{for some }\beta >0. $$

*Then*, *for* $2\le q\le \infty $, $m\ge 2n$, $\delta \in (0,1/2]$, *we have*

3.4 $$ \lambda_{n,\delta }\bigl(D:\mathbb{R}^{m}\rightarrow l^{m}_{q},\gamma_{m}\bigr) \ll \biggl(\frac{C(m,\beta)}{n+1} \biggr)^{\frac{1}{\beta }} \textstyle\begin{cases} (m^{1/q}+\sqrt{\ln (1/\delta)}, &2\le q< \infty, \\ \sqrt{\ln m+\ln (1/\delta)}, &q=\infty. \end{cases} $$

Let $\xi_{j}=\cos \theta_{j}$, $1\le j\le 2n$, denote the zeros of the Jacobi polynomial $P_{2n}^{(\alpha,\beta)}(t)$, ordered so that

$$0=:\theta_{0}< \theta_{1}< \cdots < \theta_{2n}< \theta_{2n+1}:=\pi. $$

Let $\lambda_{2n}(t)$ be the Christoffel function and $b_{j}=\lambda _{2n}(\xi_{j})$. Denote

$$W(n;\xi_{j})=\bigl(1-x+n^{-2}\bigr)^{\alpha +\frac{1}{2}} \bigl(1-x+n^{-2}\bigr)^{\beta + \frac{1}{2}}. $$

It is well known uniformly (see [15])

$$\theta_{j+1}-\theta_{j}\asymp n^{-1},\qquad \theta_{j}\asymp jn^{-1} \quad (1 \le j\le 2n), $$

and also

$$b_{j}\asymp n^{-1}w_{\alpha,\beta }(\xi_{j}) \bigl(1-\xi_{j}^{2}\bigr)^{1/2} \asymp n^{-1}W(n;\xi_{j}), $$

where the constants of equivalence depend only on *α*, *β* (see [16] or [17]).

The following lemma is well known as Gaussian quadrature formulae.

### Lemma 3.3

(See [8])

*For each* $n\ge 1$, *the quadrature*

3.5 $$ \int_{-1}^{1} f(x)w_{\alpha,\beta }(x)\,dx\asymp \sum _{j=1}^{2n}b _{j} f(\xi_{j}) $$

*is exact for all polynomials of degree* $4n-1$. *Moreover*, *for any* $1\le p\le \infty$, $f\in \Pi_{n}$, *we have*

3.6 $$ \Vert f\Vert _{p,\alpha,\beta }\asymp \Biggl(\sum _{j=1}^{2n}b_{j} \bigl\vert f(\xi_{j})\bigr\vert ^{p} \Biggr)^{1/p}. $$

An equivalence like (3.6) is generally called a Marcinkiewicz-Zygmund type inequality.

### Lemma 3.4

(See [12], Lemma 2.7)

*Let* $\alpha,\beta >-1/2$, $\sigma \in (0,\frac{1}{2\max \{\alpha,\beta \}+1})$ *and let* $b_{j}$, $1\le j\le n$, *be defined as in Lemma* 3.3. *Then*

3.7 $$ \sum_{j=1}^{n} b_{j}^{-\sigma } \ll n^{1+\sigma }. $$

Let

3.8 $$ L_{n}(x,y):=\sum_{j=0}^{\infty }\eta \biggl(\frac{j}{n}\biggr)P_{j}(x)P_{j}(y),\quad x,y\in [-1,1], $$

where $\eta \in C^{\infty }(R)$ is a nonnegative $C^{\infty }$-function on $[0,\infty)$ supported in $[0,2]$ with the properties that $\eta (t)=1$ for $0\leq t\leq 1$ and $\eta (t)>0$ for $t\in [0,2)$. For any $f\in L_{2,\alpha,\beta }$, we define

3.9 $$ \delta_{1}(f)=S_{2}(f),\qquad \delta_{k}(f)=S_{2^{k}}(f)-S_{2^{k-1}}(f) \quad \mbox{for } k=2,3\ldots, $$

where $S_{n}$ is given in (2.1). Denote by

3.10 $$ M_{k}(x,y)=\sum_{l=2^{k-1}+1}^{2^{k}}P_{l}(x)P_{l}(y) $$

the reproducing kernel of the Hilbert space $L_{2,\alpha,\beta } \cap \bigoplus_{n=2^{k-1}+1}^{2^{k}}\mathbb{P}_{n}$. Then, for $x\in [0,1]$,

$$\delta_{k}(f) (x)=\sum_{l=2^{k-1}+1}^{2^{k}} \int_{-1}^{1}f(x)P_{l}(x)P _{l}(y)w_{\alpha,\beta }(y)\,dx=\bigl\langle f,M_{k}(\cdot,x)\bigr\rangle . $$

For $f\in \bigoplus_{n=2^{k-1}+1}^{2^{k}}\mathbb{P}_{n}$,

$$f(x)=\delta_{k}(f) (x)=\bigl\langle f,M_{k}(\cdot,x)\bigr\rangle . $$

By Lemma 3.3, there exists a sequence of positive numbers $w_{i}=b _{i}\asymp n^{-1}W_{\alpha,\beta }(n;\xi_{i})$, $1\le i\le 2^{k+1}$, for which the following quadrature formula holds for all $f \in \Pi_{2^{k+3}-1}$:

3.11 $$ \int_{-1}^{1} f(t)W_{\alpha,\beta }(t)\,dt=\sum _{i=1}^{2^{k+1}}w_{i} f(\xi_{i}). $$

Moreover, for any $1\le p\le \infty$, $f\in \Pi_{2^{k}}$, we have

$$\Vert f\Vert _{p,\alpha,\beta }\asymp \Biggl(\sum _{i=1}^{2^{k+1}}w_{i} \bigl\vert f(\xi_{i})\bigr\vert ^{p} \Biggr)^{1/p}=\bigl\Vert U_{n}(f)\bigr\Vert _{\ell_{p,w}^{2^{k+1}}}, $$

where $w=(w_{1},\dots,w_{2^{k+1}})$, $U_{k}:\Pi_{2^{k}}\longmapsto \mathbb{R}^{2^{k+1}}$ is defined by

3.12 $$ U_{k}(f)=\bigl(f(\xi_{1}),\dots,f(\xi_{2^{k+1}}) \bigr), $$

and for $x\in \mathbb{R}^{2^{k+1}}$,

$$\Vert x\Vert _{\ell_{p,w}^{2^{k+1}}}:=\textstyle\begin{cases} (\sum_{i=1}^{2^{k+1}} \vert x_{i}\vert ^{p}w_{i} )^{\frac{1}{p}}, &1\le p< \infty, \\ \max_{1\le i\le {2^{k+1}}} \vert x_{i}\vert , &p=\infty. \end{cases} $$

Let the operator $T_{k}:\mathbb{R}^{2^{k+1}}\longmapsto \Pi_{2^{k+1}}$ be defined by

3.13 $$ T_{k}a(x):=\sum_{i=1}^{2^{k+1}}a_{i}w_{i}L_{2^{k+1}}(x, \xi_{i}), $$

where $a:=(a_{1},\dots,a_{2^{k+1}})\in \mathbb{R}^{2^{k+1}}$. It is shown in [12] that for $1\le q\le \infty $,

3.14 $$ \Vert T_{k}a\Vert _{q,\alpha,\beta }\ll \Vert v\Vert _{\ell_{q,w}^{2^{k+1}}}. $$

For $f\in \Pi_{2^{k+1}}$, we have

$$f(x)= \int_{-1}^{1}f(y)L_{2^{k+1}}(x,y)w_{\alpha,\beta }(x,y)\,dy= \sum_{i=1}^{2^{k+1}}w_{i}f(\xi_{i})L_{2^{k+1}}(x,\xi_{i})=T_{k}U_{k}(f) (x). $$

In what follows, we use the letters $S_{k}$, $R_{k}$, $V_{k}$ to denote $u_{k}\times u_{k}$ real diagonal matrixes as follows:

3.15 $$ \begin{aligned} &S_{k}=\operatorname{diag}\bigl(w_{1}^{\frac{1}{2}},\ldots,w_{2^{k+1}}^{\frac{1}{2}}\bigr), \\ & R_{k}=\operatorname{diag}\bigl(w_{1}^{\frac{1}{q}},\ldots,w_{2^{k+1}}^{\frac{1}{q}} \bigr), \\ &V_{k}=\operatorname{diag}\bigl(w_{1}^{-\frac{1}{2}+\frac{1}{q}},\ldots,w_{2^{k+1}}^{- \frac{1}{2}+\frac{1}{q}}\bigr), \end{aligned}$$

and use the letter $R_{k}^{-1}$ to represent the inverse matrix of $R_{k}$.

### Lemma 3.5

*For any* $z=(z_{1},\ldots,z_{2^{k+1}})\in \mathbb{R}^{2^{k+1}}$, *we have*

3.16 $$ \Biggl\Vert \sum_{j=1}^{2^{k+1}}w_{j}^{\frac{1}{2}}z_{j}M_{k}(\cdot,\xi_{j})\Biggr\Vert _{2,\alpha,\beta }\ll \Vert z\Vert _{l_{2}^{2^{k+1}}}, $$

*where* $M_{k}(x,y)$ *is given in* (3.10), *and* $(\xi_{1},\ldots,\xi_{2^{k+1}})$ *is defined as above*.

### Proof

Denote by *K* the set

$$\Biggl\{ g\in \bigoplus^{2^{k}}_{j=2^{k-1}-1} \mathbb{P}_{j} : \Vert g\Vert _{2,\alpha, \beta } \leq 1 \Biggr\} . $$

Since

$$\sum^{2^{k+1}}_{j=1} w^{1/2}_{j} z_{j} M_{k}(\cdot,\xi_{j}) \in L_{2, \alpha,\beta } \cap \Biggl(\bigoplus^{2^{k}}_{j=2^{k-1}-1} \mathbb{P}_{j}\Biggr). $$

By the Riesz representation theorem and the Cauchy-Schwarz inequality, we have

$$\begin{aligned} \Biggl\Vert \sum^{2^{k+1}}_{j=1} w^{1/2}_{j} z_{j}M_{k}(\cdot, \xi_{j})\Biggr\Vert _{2,\alpha,\beta } =& \sup_{g \in K} \Biggl\vert \Biggl\langle \sum^{2^{k+1}}_{j=1} w^{1/2}_{j} z_{j} M_{k}(\cdot , \xi_{j}),g \Biggr\rangle \Biggr\vert \\ =& \sup_{g \in K} \Biggl\vert \sum^{2^{k+1}}_{j=1} w^{1/2}_{j} z_{j} g(\xi_{j}) \Biggr\vert \\ \leq & \sup_{g \in K} \Biggl(\sum^{2^{k+1}}_{j=1} \vert z_{j}\vert ^{2}\Biggr)^{1/2} \Biggl(\sum^{2^{k+1}}_{j=1} w_{j}\bigl\vert g(\xi_{j})\bigr\vert ^{2}\Biggr)^{1/2} \\ \ll & \sup_{g \in K} \Biggl(\sum^{2^{k+1}}_{j=1} \vert z_{j}\vert ^{2}\Biggr)^{1/2} \Vert g \Vert _{2,\alpha,\beta } \\ \leq& \Vert z \Vert _{l^{2^{k+1}}_{2}}. \end{aligned}$$

 □

## Proofs of main results

Before Theorem 2.1 is proved, we establish the discretization theorems which give the reduction of the calculation of the probabilistic widths.

### Theorem 4.1

*Let* $1\leq q\leq \infty$, $\sigma \in (0,1)$, *and let the sequences of numbers* $\{n_{k}\}$ *and* $\{\sigma_{k}\}$ *be such that* $0 \leq n_{k} \leq 2^{k+1}=:m_{k}$, $\sum^{\infty }_{k=1} n_{k} \leq n$, $\sigma_{k}\in (0,1)$, $\sum^{\infty }_{k=1} \sigma_{k} \leq \sigma $. *Then*

4.1 $$ \lambda_{n,\sigma }\bigl(W^{r}_{2,\alpha,\beta },\nu,L_{q,\alpha,\beta }\bigr) \leq \sum^{\infty }_{k=1}2^{-k\rho } \lambda_{n_{k},\sigma_{k}}\bigl(V _{k} :\mathbb{R}^{m_{k}} \rightarrow l^{m_{k}}_{q},\gamma_{m_{k}}\bigr). $$

### Proof

For convenience, we write

$$\lambda_{n_{k},\sigma_{k}}:=\lambda_{n_{k},\sigma_{k}}\bigl(V_{k} : \mathbb{R} ^{m_{k}} \rightarrow l^{m_{k}}_{q}, \gamma_{m_{k}}\bigr), $$

where $\gamma_{m_{k}}$ is the standard Gaussian measure in $\mathbb{R} ^{m_{k}}$. Denote by $L_{k}$ a linear operator from $\mathbb{R}^{m_{k}}$ to $\mathbb{R}^{m_{k}}$ such that the rank of $L_{k}$ is at most $n_{k}$ and

$$\gamma_{m_{k}}\bigl(\bigl\{ y\in \mathbb{R}^{m_{k}} \vert \Vert V_{k} y-L_{k}y\Vert >2 \lambda_{n_{k},\sigma_{k}} \bigr\} \bigr) \leq \sigma_{k}. $$

Then, for any $f \in W^{r}_{2,\alpha,\beta }$, by (3.8)-(3.10), (3.14) and (3.15) we have

4.2 $$\begin{aligned} \bigl\Vert \delta_{k}(f)-T_{k}R^{-1}_{k}L_{k}S_{k}U_{k} \delta_{k}(f) \bigr\Vert _{q, \alpha,\beta } =&\bigl\Vert T_{k}U_{k}\delta_{k}(f)-T_{k}R^{-1}_{k}L_{k}S_{k}U_{k} \delta_{k}(f)\bigr\Vert _{q,\alpha,\beta } \\ \leq& \bigl\Vert U_{k}\delta_{k}(f)-R^{-1}_{k}L_{k}S_{k}U_{k} \delta_{k}(f) \bigr\Vert _{l^{m_{k}}_{q,w}} \\ =&\bigl\Vert V_{k}S_{k}U_{k} \delta_{k}(f)-L_{k}S_{k}U_{k} \delta_{k}(f) \bigr\Vert _{l ^{m_{k}}_{q}}. \end{aligned}$$

Let $y=S_{k}U_{k}\delta_{k}(f)=(w^{\frac{1}{2}}_{1} \delta_{k}(f)(\xi _{1}),\ldots,w^{\frac{1}{2}}_{m_{k}} \delta_{k}(f)(\xi_{m_{k}})) \in \mathbb{R}_{m_{k}}$, for $x\in [-1,-1]$,

$$\delta_{k}(f) (x)=\bigl\langle f,M_{k}(\cdot,x)\bigr\rangle = \bigl\langle f^{(-r)},M ^{(-r,0)}_{k}(\cdot,x) \bigr\rangle _{r} = \bigl\langle f,M^{(-2r,0)}_{k}(\cdot,x)\bigr\rangle _{r}, $$

where $M^{(r_{1},0)}_{k}(x,y)$ is the $r_{1}$-order partial derivative of $M_{k}(x,y)$ with respect to the variable $x,r_{1} \in \mathbb{R}$. Since the random vector *f* in $W^{r}_{2,\alpha,\beta }$ is a centered Gaussian random vector with a covariance operator $C_{\nu }$, the vector

$$y=S_{k}U_{k}\delta_{k}(f)=\bigl(\bigl\langle f,w^{\frac{1}{2}}_{1} M^{(-2r,0)} _{k}(\cdot, \xi_{1})\bigr\rangle _{r},\ldots,w^{\frac{1}{2}}_{m_{k}} M^{(-2r,0)} _{k}(\cdot,\xi_{m_{k}})\rangle_{r} \bigr) $$

in $\mathbb{R}^{m_{k}}$ is a random vector with a centered Gaussian distribution *γ* in $\mathbb{R}^{m_{k}}$, and its covariance matrix $C_{\gamma }$ is given by

$$C_{\gamma }= \bigl(\bigl\langle C_{\nu }\bigl(w^{\frac{1}{2}}_{i} M^{(-2r,0)}_{k} (\cdot,\xi_{i}) \bigr),w^{\frac{1}{2}}_{j} M^{(-2r,0)}_{k} (\cdot, \xi_{j}) \bigr\rangle _{r} \bigr)^{m_{k}}_{i,j=1}. $$

Since for any $z = (z_{1},\ldots,z_{m_{k}}) \in \mathbb{R}^{m_{k}}$,

$$\sum^{m_{k}}_{j=1}w_{j}^{\frac{1}{2}} z_{j} M_{k}(\cdot,\xi_{j}) \in \bigoplus _{j=2^{k-1}+1}^{2^{k}}\mathbb{P}_{j}, $$

and

$$\begin{aligned} \bigl\langle C_{\nu }\bigl(w^{\frac{1}{2}}_{i} M^{(-2r,0)}_{k} (\cdot,\xi_{i})\bigr),w ^{\frac{1}{2}}_{j} M^{(-2r,0)}_{k} (\cdot, \xi_{j})\bigr\rangle _{r} =& \bigl\langle w^{\frac{1}{2}}_{i} M^{(-2r-s,0)}_{k} (\cdot, \xi_{i}),w^{ \frac{1}{2}}_{j} M^{(-2r,0)}_{k} (\cdot,\xi_{j})\bigr\rangle _{r} \\ =& \bigl\langle w^{\frac{1}{2}}_{i} M^{(-\rho,0)}_{k} (\cdot,\xi_{i}),w ^{\frac{1}{2}}_{j} M^{(-\rho,0)}_{k} (\cdot,\xi_{j})\bigr\rangle , \end{aligned}$$

by Lemma 3.5 we get

4.3 $$\begin{aligned} \int_{\mathbb{R}^{m_{k}}}(y,z)^{2} \gamma (dy) =&z C_{\gamma }z^{T} = \sum^{m_{k}}_{i,j=1} z_{i} z_{j} \bigl\langle w^{\frac{1}{2}}_{i} M^{(- \rho,0)}_{k} (\cdot,\xi_{i}),w^{\frac{1}{2}}_{j} M^{(-\rho,0)}_{k} (\cdot,\xi_{j})\bigr\rangle \\ =& \Biggl\langle \sum^{m_{k}}_{j=1} w^{\frac{1}{2}}_{j} z_{j} M^{(-\rho,0)} _{k} (\cdot,\xi_{j}),\sum^{m_{k}}_{j=1} w^{\frac{1}{2}}_{j} z_{j} M ^{(-\rho,0)}_{k} (\cdot,\xi_{j}) \Biggr\rangle \\ =& \Biggl\Vert \sum^{m_{k}}_{j=1} w^{\frac{1}{2}}_{j} z_{j} M^{(-\rho,0)}_{k}(\cdot,\xi_{j})\Biggr\Vert ^{2}_{2} \asymp 2^{-2k\rho } \Biggl\Vert \sum^{m_{k}}_{j=1}w^{\frac{1}{2}}_{j} z_{j} M_{k} (\cdot,\xi_{j})\Biggr\Vert ^{2}_{2} \\ \ll& 2^{-2k\rho }\Vert z\Vert _{l_{2}^{m_{k}}} = 2^{-2k\rho } \int_{\mathbb{R}^{m_{k}}}(y,z)^{2} \gamma_{m_{k}}(dy). \end{aligned}$$

Now we consider the subset of $W^{r}_{2,\alpha,\beta }$

$$G_{k} :=\bigl\{ f \in W^{r}_{2,\alpha,\beta } \vert \bigl\Vert \delta_{k}(f)-T_{k}R^{-1}_{k}L_{k}S_{k}U_{k} \delta_{k}(f)\bigr\Vert _{l_{q}^{m_{k}}} >2c_{1}c_{2} 2^{-k\rho } \lambda_{n_{k},\sigma_{k}} \bigr\} , $$

where $c_{1}$, $c_{2}$ are the positive constants given in (4.2), (4.3). Then by (4.2) we get

$$\begin{aligned} \nu (G_{k}) \leq &\nu \bigl(\bigl\{ f \in W^{r}_{2,\alpha,\beta } \vert \bigl\Vert V_{k}S_{k}U_{k} \delta_{k}(f)-L_{k}S_{k}U_{k} \delta_{k}(f)\bigr\Vert _{l_{q} ^{m_{k}}} >2c_{2} 2^{-k\rho } \lambda_{n_{k},\sigma_{k}} \bigr\} \bigr) \\ =&\gamma \bigl(\bigl\{ y \in \mathbb{R}^{m_{k}} \vert \Vert V_{k} y-L_{k} y\Vert _{l_{q}^{m_{k}}} >2c_{2} 2^{-k\rho } \lambda_{n_{k},\sigma_{k}} \bigr\} \bigr). \end{aligned}$$

Note that for any $t>0$, the set $\{ y \in \mathbb{R}^{m_{k}} : \Vert V_{k} y-L_{k} y \Vert _{l_{q}^{m_{k}}} \leq t \}$ is convex symmetric. It then follows by Theorem 1.8.9 in [10] and (4.3), we have

$$\begin{aligned} \nu (G_{k}) \leq & \gamma \bigl(\bigl\{ y \in \mathbb{R}^{m_{k}} : \Vert V_{k} y-L_{k} y\Vert _{l_{q}^{m_{k}}} >2c_{2} 2^{-k\rho } \lambda_{n_{k},\sigma_{k}} \bigr\} \bigr) \\ \leq & \lambda \bigl(\bigl\{ y \in \mathbb{R}^{m_{k}} : \Vert V_{k} y-L_{k} y\Vert _{l_{q}^{m_{k}}} >2c_{2} 2^{-k\rho } \lambda_{n_{k},\sigma _{k}} \bigr\} \bigr) \\ \leq & \gamma_{m_{k}} \bigl(\bigl\{ y \in \mathbb{R}^{m_{k}} : \Vert V_{k}y-L_{k} y\Vert _{l_{q}^{m_{k}}} >2 \lambda_{n_{k},\sigma_{k}} \bigr\} \bigr) \leq \sigma_{k}, \end{aligned}$$

where *λ* is a centered Gaussian measure in $\mathbb{R}^{m_{k}}$ with covariance matrix $c_{2}^{2} 2^{-2k\rho } I_{m_{k}}$. Consider $G=\bigcup^{\infty }_{k=1} G_{k}$ and the linear operator $\widetilde{T}_{n} $ on $W^{r}_{2,\alpha,\beta }$ which is given by

$$\widetilde{T}_{n} f = \sum^{\infty }_{k=1} T_{k}R^{-1}_{k}L_{k}S_{k}U _{k}\delta_{k}(f). $$

Then

$$\nu (G) = \nu \Biggl(\bigcup^{\infty }_{k=1} G_{k}\Biggr) \leq \sum^{\infty }_{k=1} \nu (G_{k}) \leq \sum^{\infty }_{k=1} \nu (\sigma_{k}) \leq \sigma, $$

and

$$\begin{aligned} \operatorname{rank}\widetilde{T}_{n} \leq& \sum^{\infty }_{k=1} \operatorname{rank}\bigl(T_{k}R^{-1}_{k}L _{k}S_{k}U_{k} \delta_{k}\bigr) \\ \leq& \sum^{\infty }_{k=1}n_{k} \leq n. \end{aligned}$$

Thus, according to the definitions of *G*, $\widetilde{T}_{n}$, and ${L_{k}}$, we obtain

$$\begin{aligned} \lambda_{n,\delta }\bigl(W^{r}_{2,\alpha,\beta },\nu,L_{q,\alpha,\beta }\bigr) =& \sup_{f \in W^{r}_{2,\alpha,\beta } \backslash G} \Vert f- \widetilde{T}_{n}f \Vert _{q,\alpha,\beta } \\ \leq &\sup_{f \in W^{r}_{2,\alpha,\beta } \backslash G} \sum^{ \infty }_{k=1} \bigl\Vert \delta_{k}(f)-T_{k}R^{-1}_{k}L_{k}S_{k}U_{k} \delta_{k}(f) \bigr\Vert _{q,\alpha,\beta } \\ \leq & \sum^{\infty }_{k=1} \sup _{f \in W^{r}_{2,\alpha,\beta } \backslash G} \bigl\Vert \delta_{k}(f)-T_{k}R^{-1}_{k}L_{k}S_{k}U_{k} \delta_{k}(f) \bigr\Vert _{q,\alpha,\beta } \\ \ll & \sum^{\infty }_{k=1} 2^{-k \rho } \lambda_{n_{k},\sigma_{k}}, \end{aligned}$$

which completes the proof of Theorem 4.1. □

Now we turn to the lower estimates. Assume that $m \geq 6$ and $b_{1}m \leq n \leq 2b_{1}m$ with $b_{1}>0$ being independent of *n* and *m*. Set $\{x_{j}\}^{N}_{j=1}\subset \{x\in [-1,1]: \vert x\vert \leq 2/3\}$ and $x_{j+1}-x_{j}=3/m$, $j=1,\ldots, N-1$. Then $M\asymp N $ and

$$\bigl\{ x\in [-1,1] : \vert x-x_{j}\vert \leq 1/m \bigr\} \cap \bigl\{ x\in [-1,1]: \vert x-x_{i}\vert \leq 1/m\bigr\} = \emptyset, \quad \mbox{if }i \neq j. $$

We may take $b_{1}>0$ sufficiently large so that $N\geq 2n$. Let $\varphi^{1}$ be a $C^{\infty }$-function on $\mathbb{R}$ supported in $[-1,1]$, and be equal to 1 on $[-2/3,2/3]$. Let $\varphi^{2}$ be a nonnegative $C^{\infty }$-function on $\mathbb{R}$ supported in $[-1/2,1/2]$, and be equal to 1 on $[-1/4,1/4]$. Define

$$\varphi_{i}(x)= \varphi^{1}\bigl(m(x-x_{i}) \bigr) - c_{i}\varphi^{2}\bigl(m(x-x_{i}) \bigr), $$

for some $c_{i}$ such that $\int^{1}_{-1} \varphi_{i}(x) W_{\alpha, \beta }(x)\,dx =0$, $i=1,\ldots,N$. Set

$$A_{N} : = \operatorname{span}\{\varphi_{1},\ldots,\varphi_{N} \} = \Biggl\{ F_{a}(x) =\sum^{N}_{j=1} a_{j} \varphi_{j}(x): a=(a_{1},\ldots,a_{N}) \in \mathbb{R} ^{N} \Biggr\} . $$

Clearly,

$$\begin{aligned}& \varphi_{j} \in W^{2}_{2,\alpha,\beta },\quad \operatorname{supp}\varphi_{j} \subset \bigl\{ x \in [-1,1] : \vert x-x_{j} \vert \leq 1/m \bigr\} \subset \bigl\{ x \in [-1,1] : \vert x\vert \leq 5/6 \bigr\} , \\& \Vert \varphi_{j}\Vert _{q,\alpha,\beta } \asymp \biggl(\int^{2/3}_{-2/3} \bigl\vert \varphi_{j}(x) \bigr\vert ^{q}\,dx \biggr)^{1/q} = \biggl(\int^{2/3}_{-2/3} \bigl\vert \varphi^{1} \bigl(m(x-x_{j})\bigr)-c_{j} \varphi^{2} \bigl(m(x-x_{j})\bigr)\bigr\vert ^{q}\,dx \biggr)^{1/q} \\& \hphantom{\Vert \varphi_{j}\Vert _{q,\alpha,\beta }} \asymp m^{-1/q},\quad 1\leq q \leq \infty, j=1,\ldots,N, \end{aligned}$$

and

$$\operatorname{supp}\varphi_{j} \cap \operatorname{supp}\varphi_{i} = \emptyset\quad (i\neq j). $$

It follows that for $F_{a}\in A_{n}$, $a=(a_{1},\ldots,a_{N}) \in \mathbb{R}^{N}$, we have

4.4 $$ \Vert F_{a}\Vert _{q,\alpha,\beta }\asymp \Biggl(m^{-1} \sum^{N}_{j=1} \vert a_{j} \vert ^{q}\Biggr)^{1/q} = m^{-1/q}\Vert a\Vert _{l^{N}_{q}}. $$

For a nonnegative integer $\nu =0,1,\ldots $ , and $F_{a} \in A_{N}$, $a=(a_{1},\ldots,a_{N}) \in \mathbb{R}^{N} $, it follows from the definition of $-D_{\alpha,\beta } $ that

$$\operatorname{supp}(-D_{\alpha,\beta })^{\nu }(\varphi_{j})\subset \bigl\{ x \in [-1,1] : \vert x-x_{j}\vert \leq 1/m \bigr\} $$

and

$$\bigl\Vert (D_{\alpha,\beta })^{\nu }(\varphi_{j})\bigr\Vert _{q,\alpha,\beta } \leq m^{2\nu -1/q}. $$

Hence, for $1 \leq q\leq \infty $ and $F_{a}= \sum^{N}_{j=1}a_{j} \varphi_{j} \in A_{N}$,

$$\bigl\Vert (-(D_{\alpha,\beta })^{\nu }(F_{a})\bigr\Vert _{q,\alpha,\beta } \leq m ^{2\nu -1/q} \Vert a\Vert _{l^{N}_{q}}. $$

It then follows by the Kolmogorov type inequality (see Theorem 8.1 in [18]) that

4.5 $$\begin{aligned} \bigl\Vert F^{(\rho)}_{a}\bigr\Vert _{q,\alpha,\beta } =& \bigl\Vert (-D_{\alpha,\beta })^{\rho /2}(F_{a}) \bigr\Vert _{q,\alpha,\beta } \\ \ll& \bigl\Vert (-D_{\alpha,\beta })^{1+[\rho ]}(F_{a}) \bigr\Vert ^{\frac{\rho }{2+2[\rho ]}}_{q,\alpha,\beta } \Vert F_{a}\Vert ^{1-\frac{\rho }{2+2[\rho ]}}_{q,\alpha,\beta } \\ \ll& m^{\rho -1/q} \Vert a\Vert _{l^{N}_{q}} \ll m^{\rho } \Vert F_{a}\Vert _{q,\alpha,\beta }. \end{aligned}$$

For $f\in L_{1,\alpha,\beta }$ and $x \in [-1,1]$, we define

$$P_{N}(f) (x)=\sum^{N}_{j=1} \frac{\varphi_{j}(x)}{\Vert \varphi_{j}\Vert ^{2} _{2,\alpha,\beta }} \int^{1}_{-1} f(y)\varphi_{j}(y)W_{\alpha, \beta }(y)\,dy $$

and

$$Q_{N}(f) (x)=\sum^{N}_{j=1} \frac{\varphi_{j}(x)}{\Vert \varphi_{j}\Vert ^{2} _{2,\alpha,\beta }} \int^{1}_{-1} f(y)\varphi^{(\rho)}_{j}(y)W_{ \alpha,\beta }(y)\,dy. $$

Clearly, the operator $P_{N}$ is the orthogonal projector from $L_{2,\alpha,\beta }$ to $A_{N}$, and if $f\in W_{2,\alpha,\beta } ^{\rho }$, then $Q_{N}(f)(x)=P_{N}(f^{\rho })(x)$. Also, using the method in [19], we can prove that $P_{N}$ is the bounded operator from $L_{q,\alpha,\beta }$ to $A_{N}\cap L_{q,\mu }$ for $1\leq q \leq \infty $,

4.6 $$ \bigl\Vert P_{N}(f)\bigr\Vert _{q,\alpha,\beta }\ll \Vert f \Vert _{q,\alpha,\beta }. $$

Since $Q_{N}(f)\in A_{N}$ for $f\in W_{2,\alpha,\beta }^{\rho }$, we have

4.7 $$ \bigl\Vert Q_{N}(f)^{(\rho)}\bigr\Vert _{2,\alpha,\beta }\ll m^{\rho }\bigl\Vert Q_{N}(f))\bigr\Vert _{2,\alpha,\beta } =m^{\rho }\bigl\Vert P_{N}(f)^{(\rho)}\bigr\Vert _{2,\alpha, \beta }\ll m^{\rho }\bigl\Vert f^{(\rho)}\bigr\Vert _{2,\alpha,\beta }. $$

### Theorem 4.2

*Let* $1\le q\le \infty $, $\delta \in (0,1)$, *and let* *N* *be given above*. *Then*

$$\lambda_{n,\delta }\bigl(W^{r}_{2,\alpha,\beta },\nu,L_{q,\alpha,\beta }\bigr)\gg n^{1/2-\rho -1/q}\lambda_{n,\delta } \bigl(I_{N}:\mathbb{R}^{N}\rightarrow l^{N}_{q}, \gamma_{N}\bigr), $$

*where* $N\asymp n$, $N\geq 2n$ *and* $\gamma_{N}$ *is the standard Gaussian measure in* $\mathbb{R}^{N}$.

### Proof

Let $T_{n}$ be a bounded linear operator on $W^{r}_{2,\alpha,\beta }$ with rank $T_{n}\leq n$ such that

$$\nu \bigl(\bigl\{ f\in W^{r}_{2,\alpha,\beta }:\Vert f-T_{n} f\Vert _{q,\alpha,\beta }>2 \lambda_{n,\delta }\bigr\} \bigr)\leq \delta, $$

where $\lambda_{n,\delta }:=\lambda_{n,\delta }(W^{r}_{2,\alpha, \beta },\nu,L_{q,\alpha,\beta })$. Note that if *A* is a bounded linear operator from $W^{r}_{2,\alpha,\beta }$ to $W^{r}_{2,\alpha, \beta }$ and from $H(\nu)$ to $H(\nu)$, then the image measure *λ* of *ν* under *A* is also a centered Gaussian measure on $W^{r}_{2,\alpha,\beta }$ with covariance

$$R_{\lambda }(f) (f)=\bigl\langle A^{*}C_{\nu }f,A^{*}C_{\nu }f \bigr\rangle _{H(\nu)}, \quad f\in W^{r}_{2,\alpha,\beta }, $$

where $C_{\nu }$ is the covariance of the measure *ν*, $H(\nu)=W ^{\rho }_{2,\alpha,\beta }$ is the Camera-Martin space of *ν*, and $A^{*}$ is the adjoint of *A* in $H(\nu)$ (see Theorem 3.5.1 of [10]). Furthermore, if the operator *A* also satisfies

$$\Vert Af\Vert _{H(\nu)}\le \Vert f\Vert _{H(\nu)}, $$

then

$$R_{\lambda }(f) (f)=\bigl\Vert A^{*}C_{\nu }f\bigr\Vert ^{2}_{H(\nu)}\le \bigl\Vert A^{*}\bigr\Vert ^{2} \Vert C_{\nu }f\Vert \le \langle C_{\nu }f,C_{\nu }f\rangle_{H(\nu)}=R_{ \nu }(f) (f). $$

By Theorem 3.3.6 in [10], we get that for any absolutely convex Borel set *E* of $W^{r}_{2,\alpha,\beta }$ there holds the inequality

$$\nu (E)\le \lambda (E). $$

Applying (4.7) we assert that

$$\bigl\Vert Q_{N}(f)\bigr\Vert _{H(\nu)}=\bigl\Vert \bigl(Q_{N}(f)\bigr)^{(\rho)}\bigr\Vert _{2,\alpha,\beta } \ll m^{\rho }\bigl\Vert f^{(\rho)}\bigr\Vert _{2,\alpha,\beta }=m^{\rho } \Vert f\Vert _{H(\nu)}. $$

Then there exists a positive constant $c_{3}$ such that

$$\biggl\Vert \frac{1}{c_{3} m^{\rho }}Q_{N}(f)\biggr\Vert _{H(\nu)} \leq \Vert f\Vert _{H(\nu)}. $$

Note that, for any $t>0$, the set $\{f\in W^{r}_{2,\alpha,\beta }:\Vert f-T_{n} f\Vert _{q,\alpha,\beta }\leq t\}$ is absolutely convex. It then follows that

$$\nu \bigl(\bigl\{ f\in W^{r}_{2,\alpha,\beta }:\Vert f-T_{n}f\Vert _{q,\alpha,\beta }< 2 \lambda_{n,\delta }\bigr\} \bigr)\leq \lambda \bigl(\bigl\{ f\in W^{r}_{2,\alpha,\beta }: \Vert f-T_{n}f\Vert _{q,\alpha,\beta }< 2\lambda_{n,\delta }\bigr\} \bigr), $$

which leads to

$$\begin{aligned}& \nu \bigl(\bigl\{ f\in W^{r}_{2,\alpha,\beta }:\Vert f-T_{n}f\Vert _{q,\alpha,\beta }>2 \lambda_{n,\delta }\bigr\} \bigr) \\& \quad \geq \nu \bigl(\bigl\{ f\in W^{r}_{2,\alpha,\beta }:\Vert Q_{N} f - T_{n} Q_{N} f\Vert _{q,\alpha,\beta }>2 c_{3} m^{\rho } \lambda_{n,\delta } \bigr\} \bigr). \end{aligned}$$

Let $L_{N} : \mathbb{R}^{N} \rightarrow A_{N}$ and $J_{N} : A_{N} \rightarrow \mathbb{R}^{N}$ be defined by

$$L_{N}(a) (x)=\sum^{N}_{i=1} \frac{a_{i} \varphi_{i}(x)}{\Vert \varphi_{i}\Vert _{2,\alpha,\beta }}, \quad a=(a_{1},\ldots,a_{N})\in \mathbb{R}^{N} $$

and

$$J_{N}(F_{a})=\bigl(a_{1} \Vert \varphi_{1}\Vert _{2,\alpha,\beta },\ldots,a_{N} \Vert \varphi_{N}\Vert _{2,\alpha,\beta }\bigr),\quad F_{a} \in A_{N}. $$

We see at once that $L_{N}J_{N}(F_{a})=F_{a}$ for any $F_{a}\in A_{N}$. Set $y=(y_{1},\ldots,y_{N})\in \mathbb{R}^{N} $, where $y_{j} = \frac{1}{ \Vert \varphi_{j}\Vert _{2,\alpha,\beta }}\langle f, \varphi_{j}^{(\rho)} \rangle $. Then $y=J_{N} Q_{N} (f)$. Thus by (4.4) and $\Vert \varphi_{j}\Vert _{2,\alpha,\beta }\asymp m^{-\frac{1}{2}}$, we obtain

4.8 $$ \bigl\Vert L_{N}(a)\bigr\Vert _{q,\alpha,\beta }\asymp m^{-\frac{1}{q}+\frac{1}{2}}\Vert a\Vert _{ l_{q}^{N}}. $$

Combining (4.6) with (4.8), we conclude that for any $f\in W^{r}_{2, \alpha,\beta }$,

$$\begin{aligned} \bigl\Vert Q_{N}(f)-T_{N}Q_{N} (f)\bigr\Vert _{q,\alpha,\beta } \gg &\bigl\Vert P_{N}\bigl(Q_{N}(f) \bigr)-P_{N} T_{n}Q_{N}(f)Q\bigr\Vert _{q,\alpha,\beta } \\ =&\bigl\Vert L_{N}J_{N}Q_{N}(f)-L_{N}J_{N}P_{N}T_{N}L_{N}J_{N}Q_{N}(f) \bigr\Vert _{q, \alpha,\beta } \\ \gg & m^{-\frac{1}{q}+\frac{1}{2}} \bigl\Vert J_{N}Q_{N}(f)-J_{N}P_{N}T_{n}L_{N}J_{N}Q_{N}(f) \bigr\Vert _{l^{N}_{q}} \\ \gg & m^{-\frac{1}{q}+\frac{1}{2}} \Vert y-J_{N}P_{N}T_{n}L_{N}y \Vert _{l ^{N}_{q}}. \end{aligned}$$

Remark that $g_{k}=\frac{\varphi_{k}}{\Vert \varphi_{k}\Vert _{2,\alpha, \beta }}$, $k=1,2,\ldots,N$, is an orthonormal system in $L_{2,\alpha,\beta }$ and $g_{k}\in H(v)=W^{\rho }_{2,\alpha,\beta }$. Then the random vector $(\langle f,g^{(\rho)}_{1}\rangle,\ldots,\langle f,g ^{(\rho)}_{N}\rangle)=y$ in $\mathbb{R}^{N}$ on the measurable space $(W^{r}_{2,\alpha,\beta },\nu)$ has the standard Gaussian distribution $r_{N}$ in $\mathbb{R}^{N}$. It then follows that

$$\begin{aligned}& \nu \bigl(\bigl\{ f\in W^{r}_{2,\alpha,\beta }:\bigl\Vert Q_{N}(f)-T_{n}Q_{N}(f)\bigr\Vert _{q, \alpha,\beta }>2c_{3}m^{\rho }\lambda_{n,\delta }\bigr\} \bigr) \\& \quad \geq \nu \bigl(\bigl\{ f\in W^{r}_{2,\alpha,\beta }:\Vert y-T_{J}NP_{N}T_{n}L_{N}y\Vert _{l^{N}_{q}} >c_{4}m^{\rho +\frac{1}{q}-\frac{1}{2}} \lambda_{n, \delta }\bigr\} \bigr) \\& \quad =r_{N}\bigl(\bigl\{ y\in \mathbb{R}^{N}:\Vert y-T_{J}NP_{N}T_{n}L_{N}y\Vert _{l^{N}_{q}} >c _{4}m^{\rho +\frac{1}{q}-\frac{1}{2}} \lambda_{n,\delta }\bigr\} \bigr) \\& \quad =:r_{N}(G), \end{aligned}$$

where $c_{4}$ is a positive constant. Clearly, $\operatorname{rank}(J_{N}P_{N}T_{n}L _{N})\leq n$ and

$$r_{N}(G)\leq \nu \bigl(\bigl\{ f\in W^{r}_{2,\alpha,\beta }: \Vert f-T_{n}f\Vert _{q, \alpha,\beta }>2\lambda_{n,\delta }\bigr\} \bigr) \leq \delta. $$

Consequently,

$$\begin{aligned} \lambda_{n,\delta }\bigl(I_{N}:\mathbb{R}^{N} \rightarrow l^{N}_{q},r_{N}\bigr) =& \inf _{G} \inf_{I_{N}} \sup_{x\in \mathbb{R}^{N}\setminus G} \Vert I_{N}x-T_{n}x\Vert _{l^{N}_{q}} \\ \leq & \sup_{y\in \mathbb{R}^{N}\setminus G}\Vert I_{N}y-J_{N}P_{N}T_{n}L_{N}y \Vert _{l^{N}_{q}} \\ \ll & m^{\rho +\frac{1}{q}-\frac{1}{2}}\lambda_{n,\delta }, \end{aligned}$$

which implies

$$\begin{aligned} \lambda_{n,\delta }\bigl(W^{r}_{2,\alpha,\beta },\nu,L_{q,\alpha,\beta }\bigr) \ll & m^{-\rho -\frac{1}{q}+\frac{1}{2}}\lambda_{n,\delta } \bigl(I_{N}: \mathbb{R}^{N}\rightarrow l^{N}_{q},r_{N} \bigr) \\ \asymp & n^{-\rho -\frac{1}{q}+\frac{1}{2}}\lambda_{n,\delta }\bigl(I_{N}: \mathbb{R}^{N}\rightarrow l^{N}_{q},r_{N} \bigr). \end{aligned}$$

This completes the proof of Theorem 4.2. □

Now, we are in a position to prove Theorem 2.1.

### Proof

For the lower estimates, using Theorem 4.2 and Lemma 3.1, we have for $1\le q\le 2$

$$\begin{aligned} \lambda_{n,\delta }\bigl(W_{2,\alpha,\beta }^{r},\nu,L_{q,\alpha,\beta }\bigr) \gg & n^{-\rho +1/2-1/q}\lambda_{n,\delta } \bigl(I_{N}:\mathbb{R}^{N} \rightarrow l_{q}^{N}, \gamma_{N}\bigr) \\ \asymp & n^{-\rho +1/2-1/q} \biggl(N^{1/q}+N^{1/q-1/2}\biggl(\ln \biggl(\frac{1}{ \delta }\biggr)\biggr)^{1/2} \biggr) \\ \asymp & n^{1/2-\rho } \biggl(1+n^{-1/2}\biggl(\ln \biggl(\frac{1}{\delta }\biggr)\biggr)^{1/2} \biggr). \end{aligned}$$

For $2\le q< \infty $, we have

$$\begin{aligned} \lambda_{n,\delta }\bigl(W_{2,\alpha,\beta }^{r},\nu,L_{q,\alpha,\beta }\bigr) \gg & n^{-\rho +1/2-1/q} \biggl(n^{1/q}+\biggl(\ln \biggl(\frac{1}{\delta }\biggr)\biggr)^{1/2} \biggr) \\ \asymp & n^{1/2-\rho } \biggl(1+n^{-1/q}\biggl(\ln \biggl(\frac{1}{\delta }\biggr)\biggr)^{1/2} \biggr). \end{aligned}$$

And for $q=\infty $,

$$\begin{aligned} \lambda_{n,\delta }\bigl(W_{2,\alpha,\beta }^{r},\nu,L_{q,\alpha,\beta }\bigr) \gg & n^{-\rho +1/2-1/q} \biggl(\ln m+\ln \biggl(\frac{1}{\delta }\biggr) \biggr)^{1/2} \\ =& n^{1/2-\rho } \biggl(\ln \biggl(\frac{m}{\delta }\biggr) \biggr)^{1/2} . \end{aligned}$$

It remains to prove the upper estimates. For $2\le q\le \infty $ and any fixed natural number *n*, assume $C_{1}2^{m}\le n\le C_{1}^{2}2^{m}$ with $C_{1}>0$ to be specified later. We may take sufficiently small positive numbers $\varepsilon >0$ such that $\rho >\frac{1}{2}+(1+ \varepsilon)(2\max \{\alpha,\beta \}+1+\varepsilon)(\frac{1}{2}- \frac{1}{q})$. Set

$$n_{j}=\textstyle\begin{cases} 2^{j+1}, & \mbox{if } j\le m, \\ 2^{j+1}2^{(1+\varepsilon)(m-j)-1}, & \mbox{if } j> m, \end{cases} $$

and

$$\delta_{j}=\textstyle\begin{cases} 0, & \mbox{if } j\le m, \\ \delta 2^{m-j}, & \mbox{if } j> m. \end{cases} $$

Then

$$\sum_{j\ge 0}n_{j}\ll \sum _{j\le m}2^{j}+\sum_{j>m}2^{m(1+\varepsilon)-\varepsilon j} \ll 2^{m} $$

and

$$\sum_{j\ge 0}\delta_{j}=\delta \sum _{j\le m}2^{m-j}\le \delta. $$

Thus, we can take $C_{1}$ sufficiently large so that

$$\sum_{j=0}^{\infty }n_{j}\le C_{1}2^{m}\le n. $$

It follows from Lemma 3.4 for $\tau \in (0,\frac{1}{(2\max \{\alpha, \beta \}+1)(1/2-1/q)})$, $2\le q\le \infty $,

$$\sum_{j=1}^{n} b_{j}^{-\tau (1/2-1/q)} \ll 2^{k[1+\tau (1/2-1/q)]}=2^{k+k \tau (1/2-1/q)}. $$

If $j\le m$, then $n_{j}=2^{j+1}$, and thence $\lambda_{n_{j},\delta _{j}}(V_{j}:\mathbb{R}^{2^{j+1}}\rightarrow l_{q}^{2^{j+1}},\gamma_{2^{j+1}})=0$. If $j>m$, then taking $\frac{1}{\tau }=(2\max \{\alpha,\beta \}+1+ \varepsilon)(1/2-1/q)$ and applying Lemma 3.2, Theorem 4.1, we obtain for $2\le q<\infty $,

$$\begin{aligned}& \lambda_{n_{j},\delta_{j}}\bigl(V_{j}:\mathbb{R}^{2^{j+1}} \rightarrow l_{q} ^{2^{j+1}},\gamma_{2^{j+1}}\bigr) \\& \quad \ll \biggl(\frac{C(m,\tau)}{n_{j}+1} \biggr)^{1/ \tau } \biggl(2^{(j+1)/q}+\sqrt{\ln \frac{1}{\delta }} \biggr) \\& \quad \ll 2^{j(1/2-1/q)}2^{-(1+\varepsilon)(m-j)(2\max \{\alpha,\beta \}+1+\varepsilon)(\frac{1}{2}-\frac{1}{q})} \biggl(2^{\frac{j}{q}}+\sqrt{ \ln \frac{1}{\delta }} \biggr), \end{aligned}$$

which yields

4.9 $$\begin{aligned}& \lambda_{n,\delta }\bigl(W_{2,\alpha,\beta }^{r},\nu,L_{q,\alpha,\beta }\bigr) \\& \quad \ll \sum_{j=m+1}^{\infty }2^{-j\rho }2^{j(1/2-1/q)}2^{-(1+\varepsilon)(m-j)(2\max \{\alpha,\beta \}+1+\varepsilon)(\frac{1}{2}- \frac{1}{q})}2^{1/2-1/q} \biggl(2^{\frac{j}{q}}+\sqrt{\ln \frac{1}{ \delta }} \biggr) \\& \quad \ll 2^{-m(\rho -\frac{1}{2}+\frac{1}{q})} \biggl(2^{\frac{m}{q}}+\sqrt{ \ln \frac{1}{\delta }} \biggr) \asymp n^{1/2-\rho } \biggl(1+n^{-1/q}\sqrt{ \ln \frac{1}{\delta }} \biggr). \end{aligned}$$

For $q=\infty $, by Lemma 3.2 we get

$$\begin{aligned} \lambda_{n_{j},\delta_{j}}\bigl(V_{j}:\mathbb{R}^{2^{j+1}} \rightarrow l_{q} ^{2^{j+1}},\gamma_{2^{j+1}}\bigr) \ll & \biggl(\frac{C(2^{j+1},\tau)}{n_{j}+1} \biggr)^{1/\tau }\sqrt{\ln 2^{j+1}+ \ln \frac{1}{\delta }} \\ =&2^{j/2-(1+\varepsilon)(m-j)(2\max \{\alpha,\beta \}+1+\varepsilon)/2}\sqrt{j+\ln \frac{1}{\delta }}, \end{aligned}$$

then applying Theorem 4.1, we obtain

4.10 $$\begin{aligned} \lambda_{n,\delta }\bigl(W_{2,\alpha,\beta }^{r},\nu,L_{\infty,\alpha, \beta }\bigr) &\ll \sum_{j=m+1}^{\infty }2^{-j\rho }2^{j/2-(1+\varepsilon)(m-j)(2 \max \{\alpha,\beta \}+1+\varepsilon)/2} \sqrt{j+\ln \frac{1}{ \delta }} \\ &\ll 2^{-m(\rho -\frac{1}{2})}\sqrt{m+\ln \frac{1}{\delta }} \asymp n^{1/2-\rho } \sqrt{\ln \frac{n}{\delta }}. \end{aligned}$$

To finish the proof of the upper estimates, we only need to show that, for $1\le q<2$,

$$\lambda_{n,\delta }\bigl(W_{2,\alpha,\beta }^{r},\nu,L_{q,\alpha,\beta }\bigr)\ll \lambda_{n,\delta }\bigl(W_{2,\alpha,\beta }^{r}, \nu,L_{2,\alpha, \beta }\bigr) \ll n^{1/2-\rho } \biggl(1+n^{-1}\sqrt{ \ln \frac{1}{\delta }} \biggr)^{1/2}. $$

Theorem 2.1 is proved. □

## Conclusions

In this paper, optimal estimates of the probabilistic linear $(n,\delta)$-widths of the weighted Sobolev space $W_{2,{\alpha, \beta }}^{r}$ on $[-1,1]$ are established. This kind of estimates play an important role in the widths theory and have a wide range of applications in the approximation theory of functions, numerical solutions of differential and integral equations, and statistical estimates.
